# Translations from Foreign Journals

**Published:** 1856-07

**Authors:** Ch. F. J. Lehlbach

**Affiliations:** Newark, N. J.


*Translations from Foreign Journals.* By CH. F. J. LEHLBACH, M. D., Newark, N. J.

*Experiments on the body of a decapitated criminal* by the Professors, H. Müller, Kölliker, and Virchow, at the University of Wurzburg (Wurzburg, Verhandl., 1854, v. i.)

On irritation of the sympathetic nerve, one inch above the superior cervical ganglion, the pupil speedily dilated, and the dilatation lasted as long as the irritation was continued. The same result was obtained by irritation of the pneumogastric nerve. The presence of air was determined in the superficial cerebral nerves. Irritation of the oculo-motory nerve did not produce any effect upon the pupil. The centre of the yellow spot of the retina (macula lutea s. flava retinæ) was distinguished by great transparency. The cavity of the nostrils has ciliary motion throughout, even in the olfactory region of the cribriform bone, which, according to Kölliker, Todd and Bowman, should possess a peculiar lining membrane, *not* ciliary. The cavernous tissue, discovered at the inferior concha by Kölliker, showed contractions on the application of Duchennne's electrical apparatus, and it was found to possess a large number of nonstriated muscular fibres. Neither the tympanum nor the auditory ossicula possess ciliary motion; it is, however, present throughout the promontory of the tympanum. In the ligamentum spinale of the cochlea were found a number of *corpuscula amylacea.* Of the ventricles of the brain only the second manifested distinct ciliary motion; it was absent at the choroid plexuses. On the application of an electrical current the venous coats are thrown into transverse and longitudinal folds, as are also the lacteals, which discharged the chyle in a small stream, while under the influence of the electric current. The electrical current produced but feeble contraction of the coats of the intestines and intestinal villi. The spleen (under the elect. current) furrowed itself, and the furrows thus produced on its surface corresponded exactly to the course of insertion of the processes of the fibrous or proper covering of the organ (trabecules of the spleen.) The galvanic current produced erection of the nipple, and contractions of the corpora cavernosa penis. The contractions of the latter were observed on pieces separated from the body. (Temperature of the body while experimented upon, 28–29° R., or 95–97° F.)

*Division of Nerves in Traumatic Tetanus.*—In his inaugural dissertation, "Nonnulla de nervorum sectione ad tetanum traumaticum sanandam Instituta, Lipsiac, 1854," Dr. J. Kuehn quotes eight cases of traumatic tetanus in which division of the nerve was followed with success, and to these cases he adds a ninth, which he had occasion to obse. ve at Leipsic, with Prof. G. B. Guenther. A lad, aged 13, while playing, fell from a pump, so as to receive a complicated fracture of both bones of the forearm, together with a luxation of the elbow-joint. Four weeks afterwards, he was admitted into the Leipsic Hospital. On the day after his admission, mortification of the fingers took place, which gradually extended, until a line of demarkation appeared just above the wrist; the hand was removed, and the projecting bones resected. The wound cicatrized well, so that the patient was about being discharged. All of a sudden, the whole body became convulsed, the lower extremities were in a violent state of spasmodic action; the arms now flexed, now rigidly extended; the jaws could hardly be separated; the head was retracted; the muscles of the neck contracted. The patient, with all this, was conscious, his face was flushed, manifesting fear, and he complained of violent pain in the cicatrix, which was aggravated by the slightest pressure, or even by touching it. These attacks returned in irregular intervals. The circumstance, that the pain was definitely fixed in the cicatrix, led easily to the conclusion, that the tetanic spasms were caused by an irritation of the median nerve, which corresponded to the situation. After the spasms had lasted for two hours, the division of this nerve was determined upon. By a longitudinal incision the nerve, which, though of normal circumference, had been transformed into a callous mass, was exposed, a second, transverse incision separated the soft parts down to the bone, and a portion of the nerve 4–5‴ long was extirpated. The operation was very painful, and very bloody, on account of the impossibility of ligating the arteria interossea separately in the hard, indurated mass. The spasmodic attacks at once abated; one-fourth of a grain of the morph. acet. put the patient to sleep, and twelve days after the operation he could be discharged. Three months afterwards our author saw the lad again. He had been the subject of three attacks of an epileptic kind, probably the result of a new neuritis induced in the new cicatrix. (Our author calls these epileptic attacks "a *mild* (or chronic) form of tetanus.")

Notwithstanding these unfavorable subsequent incidents, the author believes himself justified in adding this case to the number of successful cases, and points to the fact, that in all recorded (by him ?) cases the section of the nerve was made in the first forty-eight hours. To this early interference must be attributed in his opinion the successful issue, because the operation being longer delayed, the brain itself would suffer such changes by the continued irritation, as to become itself the cause of a continuance of the tetanus, even after division of the nerve had been accomplished.

The other consequences of the operation, loss of sensibility and motion, the author thinks are not to be taken into consideration, as in tetanus our indication is a "*vital*" one, and the loss of sensibility is in most cases but temporary, while that of motion is often not to be taken into account at all, on account of amputation having previously been resorted to. As regards Guérin's and Bonnet's subcutaneous section, it is difficult, often impracticable, and always insecure in its effect.

*Physiological Effects of Aconitin.*—From a large number of experiments performed on animals, in reference to the pharmacodynamic effects, and therapeutical application of *Aconitin*, Leonidas Van Praeg (Virchow's *Archiv.*) has obtained the following result:

*Aconitin* exercises a retarding influence upon respiration, has a paralyzing effect upon the voluntary muscular system; its action upon the brain is depressing. It does not act upon the circulation as a depressant, but under its administration the pulse becomes fluctuating, manifesting great differences. As a general rule, aconitin produces dilatation of the pupils; less constantly, increased salivary and urinary secretions. In cases where death takes place suddenly from the administration of aconitin, it is death by *asphyxia;* where it takes place after the lapse of some time, the animals experimented upon seem to die away, as it were, exhausted. The administration of the extract. aconit. causes much more severe intestinal irritation than the administration of the alkaloid; in most other points their effects are parallel. In a pharmacodynamic point of view, aconitin is by far to be preferred to the extract, because, if chemically pure, it does not act as a local acrid irritant to the intestines, like the well-prepared extract; and because it can always be obtained of the same strength.

The largest dose, which can be administered without danger, may be stated as three-fourths of a grain. *Turnbull* states to have seen good results from its external application in neuralgias and rheumatic affections. *Skey* gave an ointment of aconitin gr. v, cerat. ℥vj, in three cases of tic doulenreux with good results. *Broches* used a salve of aconitin, gr. ij; epis. vin. q. s. ut. salv. axung. porc. ℥ij, in neuralgias of the fifth pair. After a few applications, the frequency and intensity of the attacks diminished, and after six weeks' continuance of the treatment, the disease was suspended, after having been treated for years with belladonna, veratrine, strychnine, and iodine unsuccessfully. The following are the author's conclusions:

1. That aconitin acts in most points like the extracts, aconit., alcoh., and is therefore to be employed in the same diseases.

2. It is far preferable to every other preparation of the article, on account of the unvariableness of the alkaloid, when properly prepared; while the plants, according to their different place of growth, and according to the year in which they have been collected, always show immense differences in their action.

3. The aconitin is devoid of the acrid, irritating nature of the extract.

[*Reporter*.

